# TNF-α Triggers RIP1/FADD/Caspase-8-Mediated Apoptosis of Astrocytes and RIP3/MLKL-Mediated Necroptosis of Neurons Induced by *Angiostrongylus cantonensis* Infection

**DOI:** 10.1007/s10571-021-01063-w

**Published:** 2021-03-08

**Authors:** Hongli Zhou, Minyu Zhou, Yue Hu, Yanin Limpanon, Yubin Ma, Ping Huang, Paron Dekumyoy, Wanchai Maleewong, Zhiyue Lv

**Affiliations:** 1 Key Laboratory of Tropical Disease Control (Sun Yat-Sen University), Ministry of Education, Guangzhou, China; 2 NHC Key Laboratory of Control of Tropical Diseases, Hainan Medical University, Haikou, China; 3 Department of Laboratory Medicine, The First Affiliated Hospital, Hainan Medical University, Haikou, China; 4 Faculty of Tropical Medicine, Mahidol University, Bangkok, Thailand; 5 Faculty of Medicine, Khon Kaen University, Khon Kaen, Thailand

**Keywords:** TNF-α, *Angiostrongylus cantonensis*, Apoptosis, Necroptosis

## Abstract

*Angiostrongylus cantonensis* (AC) can cause severe eosinophilic meningitis or encephalitis in non-permissive hosts accompanied by apoptosis and necroptosis of brain cells. However, the explicit underlying molecular basis of apoptosis and necroptosis upon AC infection has not yet been elucidated. To determine the specific pathways of apoptosis and necroptosis upon AC infection, gene set enrichment analysis (GSEA) and protein–protein interaction (PPI) analysis for gene expression microarray (accession number: GSE159486) of mouse brain infected by AC revealed that TNF-α likely played a central role in the apoptosis and necroptosis in the context of AC infection, which was further confirmed via an in vivo rescue assay after treating with TNF-α inhibitor. The signalling axes involved in apoptosis and necroptosis were investigated via immunoprecipitation and immunoblotting. Immunofluorescence was used to identify the specific cells that underwent apoptosis or necroptosis. The results showed that TNF-α induced apoptosis of astrocytes through the RIP1/FADD/Caspase-8 axis and induced necroptosis of neurons by the RIP3/MLKL signalling pathway. In addition, in vitro assay revealed that TNF-α secretion by microglia increased upon LSA stimulation and caused necroptosis of neurons. The present study provided the first evidence that TNF-α was secreted by microglia stimulated by AC infection, which caused cell death via parallel pathways of astrocyte apoptosis (mediated by the RIP1/FADD/caspase-8 axis) and neuron necroptosis (driven by the RIP3/MLKL complex). Our research comprehensively elucidated the mechanism of cell death after AC infection and provided new insight into targeting TNF-α signalling as a therapeutic strategy for CNS injury.

## Background

*Angiostrongylus cantonensis* (hereinafter referred to as AC), also known as rat lungworm, is a metastrongylid pathogen and was first identified in 1935 in Guang Zhou, China (Chen 1935; Hu et al. 2018). After that, additional cases have been reported, especially in underdeveloped countries and territories such as Thailand (Apichat et al. 2016), Oceania and numerous Pacific islands (Lv et al. 2017). AC infection remains the main causative agent of eosinophilic meningitis worldwide (Flerlage et al. 2017). As non-permissive hosts of AC (Ji et al. 2017; Zhou et al. 2019), mice and human beings could be infected by eating raw or uncooked snails containing third-stage larvae, which causes severe eosinophilic meningitis or encephalitis (Mengying et al. 2017). Patients infected by AC suffered from a drastic inflammatory response in the host, neurological impairment and neurodegenerative lesions (Yan et al. 2018; Yoshida et al. 1996), finally leading to memory and cognitive deterioration as a result of the impaired function and irreversible cell death of brain cells (Mengying et al. 2017). To date, the treatment for angiostrongyliasis mainly relies on broad-spectrum antiparasitic drugs such as albendazole and treatment of the symptoms. However, due to a lack of specificity, the pathological outcomes of nasty angiostrongyliasis cannot be markedly improved. Hence, it is urgent to uncover the underlying mechanism of the cell death of brain cells induced by AC infection to provide a new theoretical basis for developing more effective and specific treatment methods.

Tumour necrosis factor-α (TNF-α) is one of the most important pro-inflammatory cytokines (Akash et al. 2018; Al-Gayyar and Elsherbiny 2013), is mainly secreted by macrophage-like cells and functions to regulate the processes of inflammation (Zelova and Hosek 2013), apoptosis (Naimi et al. 2018) and necroptosis (Gunther et al. 2011) by the way of the paracrine system (Lin et al. 2018; Ye et al. 2013). Parasitic infections often lead to the upregulation of TNF-α, which was observed in cases infected with various parasites including *A. cantonensis, Leishmania braziliensis* (Nieto Gomez et al. 2019; Polari et al. 2019; Schwartz et al. 2018), *Plasmodium* (Grau and Lou 1995) and *Toxoplasma gondii* (Park et al. 2019; Pego et al. 2019). As a high level of TNF-α tends to be positively correlated with poor pathological outcomes of patients, an inhibitor of TNF-α has been used to improve the symptoms in some cases (Zhou et al. 2020), indicating that TNF-α signalling could be a promising therapeutic target.

Programmed cell death is regulated by specific genes including TNF-α and sequentially activated signalling pathways, among which apoptosis and necroptosis have been well investigated. As reported, TNF-α could induce apoptosis of various cells mainly through RIP1/FADD/Caspase-8 signalling (Zheng et al. 2016) and could trigger necroptosis of cells via RIP1/RIP3/MLKL signalling (Hu et al. 2020). Many studies have demonstrated that the mechanisms of apoptosis and necroptosis are related to TNF-α in viral infections (Gyurkovska and Ivanovska 2016) and tumours (Balkwill 2006) and promote a treatment target of TNF-α for these diseases (Monaco et al. 2015); however, in parasitic diseases (Barbosa et al. 2018; Carneiro et al. 2018; de Carvalho and Zamboni 2020; Eugenin et al. 2019; Lee et al. 2019; Zamboni and Lima-Junior 2015) much is still unknown. Furthermore, AC is the only neurotropic helminth, and our previous study discovered that the cell death of a mouse brain infected by AC displayed parallel pathways of apoptosis (driven by cleaved caspase-3) and necroptosis [reliant on the host kinase receptor interacting kinase protein3 (RIP3)] (Mengying et al. 2017). However, the explicit molecular basis of apoptosis and necroptosis during AC infection remains to be elucidated.

To illustrate the underlying molecular mechanism of apoptosis and necroptosis in the brain tissue of a non-permissive host infected with AC, gene set enrichment analysis (GSEA) coupled with protein–protein interaction (PPI) network construction analyses was first applied to explore the potential pathways of apoptosis and necroptosis upon AC infection. Mice were treated with a TNF-α inhibitor to investigate the role of TNF-α in apoptosis and necroptosis of mouse brain cells induced by AC. To further depict the specific molecular basis of apoptosis and necroptosis due to AC infection, RT-qPCR, western blot, co-immunoprecipitation and immunofluorescence analyses were applied to explore the potential signalling network of apoptosis and necroptosis in mouse brains, and RT-qPCR, western blot and flow cytometry analyses were performed to detect the source of TNF-α after AC infection.

## Methods

### Animals

Twelve female BALB/c mice (6–8 weeks old, 18–20 g) were purchased from Charles River Laboratories (Beijing, China) and divided into three groups randomly and equally (4 mice for each group; normal control group: Ctrl; AC infection group: INF; AC infection combined with acetylcysteine treatment group: ACE). Twenty third-stage infectious AC larvae were counted and used to infect the INF and ACE group mice by oral gavage. Additionally, the ACE and INF group mice were, respectively, intraperitoneally injected with acetylcysteine (TNF-α inhibitor, 0.1 mg/g) and phosphate-buffered saline (PBS) daily post infection. All the mice were housed in a specific pathogen-free, temperature-controlled environment with a 12 h light/dark cycle.

### Parasites and Larvae Soluble Antigen (LSA)

The infectious third-stage AC larvae were obtained from *Biomphalaria glabrata* as previously described (Wan et al. 2018) to infect mice, and the young adult AC were harvested from mouse brains 21 days post infection (dpi), washed repeatedly to remove host cells, homogenized on ice to release the soluble antigen, centrifuged at 12,000 rpm and filtered through a 0.22 μm sterile filter (Millipore, MA, USA). The protein concentrations of LSA were quantified by a bicinchoninic acid (BCA) kit (Beyotime, Wuhan, China).

### GSEA and PPI Network

To obtain the differentially expressed genes (DEGs) between mouse brain tissues with AC infection and normal control, a gene expression array was performed and data were analysed by Limma package in R language 3.53. Then, GSEA was conducted using the clusterProfiler package (http://www.bioconductor.org/packages/release/bioc/html/clusterProfiler.html) to analyse the critical pathway enriched by AC infection. To identify the hub gene of gene sets in an enriched pathway, PPI analysis was carried out in the STRING database (https://string-db.org/) and visualized by DisGeNET app of Cytoscape software (http://apps.cytoscape.org/apps/disgenetapp) according to the instructions. All the data of the gene expression microarray involved in the present study have been deposited to GEO database with the accession number of GSE159486.

### Cell Lines

The cell lines used in this study included N9 (mouse microglia cell line) and HT22 (mouse hippocampal neuronal cell line), and N9 was preserved in the Key Laboratory of Tropical Disease Control (Sun Yat-sen University), Ministry of Education and was cultured in Dulbecco's modified Eagle Medium/Nutrient Mixture F-12 medium (Gibco, USA) supplemented with 10% foetal bovine serum (Gibco, USA) and 100 U/ml penicillin/streptomycin (Invitrogen, USA). The HT22 cell line was a kind gift from professor Jun Liu in Sun Yat-sen Memorial Hospital and was cultured with Dulbecco's modified Eagle medium (Gibco, USA) plus 10% foetal bovine serum and 100 U/ml penicillin/streptomycin. Both cell lines were cultured in a humidified atmosphere containing 5% CO_2_ at 37 °C and were tested for *Mycoplasma* contamination by RT-PCR regularly. The murine microglial cell line N9 used in this study was derived from mouse brain and was a kind gift from professor Zhongdao Wu (Department of Parasitology, Zhongshan School of Medicine, Sun Yat-sen University, Guangzhou, China).

### H&E Staining

All mice were sacrificed under anaesthetization (4% chloral hydrate, intraperitoneal injection, 0.1 ml/10 g) at 21 days post treatment and the brain tissues were resected, fixed in 4% paraformaldehyde, paraffin embedded and sectioned into 5 μm sections for H&E staining analysis according to standard procedures as previously described (Ji et al. 2017). Histological configuration of mouse brain tissue was captured, imaged and analysed under an inverted microscope (Lecia, Heidelberg, Germany).

### Quantitative Real-Time PCR

Total RNA was extracted from mouse brain tissues or cell lines using TRIzol Reagent (Invitrogen, Carlsbad, CA) and the purity and concentration of RNA was determined by NanoDrop One (Thermo Fisher scientific, Waltham, USA). The eligible RNA was subjected to a reverse transcriptase reaction with RevertAid First Strand cDNA Kit (Thermo Fisher scientific, Waltham, USA) according to the manufacturer’s instructions. Quantitative real-time PCR (RT-qPCR) reactions were amplified for 10 min at 95 °C followed by 40 cycles at 95 °C for 15 s and 60 °C for 1 min with the use of SYBR Green (TaKaRa, Dalian, China) on a LightCycler480 Real-Time PCR System (Roche Diagnostics, Reinach, Switzerland). The relative mRNA expression level of each gene was compared to that of β-actin (internal control) by the 2^−ΔΔCt^ method. The primers used in this study are listed in Table 1.

**Table 1:** Primers for qPCR used in this study

Gene symbol	Forward primer sequence	Reverse primer sequence
Caspase-3	TGGTGATGAAGGGGTCATTTATG	TTCGGCTTTCCAGTCAGACTC
Caspase-4	TGTCATCTCTTTGATATATTCCTGAAG	CAAGGTTGCCCGATCAAT
Caspase-6	AGACAAGCTGGACAACGTGACC	CCAGGAGCCATTCACAGTTTCT
Caspase-7	AAGACGGAGTTGACGCCAAG	CCGCAGAGGCATTTCTCTTC
Caspase-8	TGCTTGGACTACATCCCACAC	TGCAGTCTAGGAAGTTGACCA
RIP3	AAGTGCAGATTGGGAACTACAACTC	AGAATGTTGTGAGCTTCAGGAAGTG
RIP1	GAAGACAGACCTAGACAGCGG	CCAGTAGCTTCACCACTCGAC
Fas	TATCAAGGAGGCCCATTTTGC	TGTTTCCACTTCTAAACCATGCT
FADD	GCGCCGACACGATCTACTG	TTACCCGCTCACTCAGACTTC
TRADD	GGCAGTGCATACCTGTTTTTG	AAATACCCCACTCTCTGACAGT
TNF-α	GAACTGGCAGAAGAGGCACT	AGGGTCTGGGCCATAGAACT
β-actin	GCTGTCCCTGTATGCCTCT	GTCTTTACGGATGTCAACG

### Immunoblotting

Mouse brain tissues were lysed in RIPA buffer (Thermo Fisher scientific, USA) plus a protease and phosphatase inhibitor cocktail (1:1000, Thermo Fisher scientific, USA) on ice for 5 min, then the lysates were centrifuged at 12,000 rpm for 15 min under 4 °C and the supernatant was preserved as total protein, the concentration of which was determined by a BCA assay. Next, total protein was denatured at 100 °C in boiling water for 5 min and 20 μg of denatured protein was subjected to SDS–polyacrylamide gel electrophoresis prior to transfer onto a polyvinylidene fluoride (PVDF, 0.22 μm) membrane (Merck Millipore, MA, USA), which was blocked with 5% non-fat milk (TBST as solvent) for 2 h at room temperature before an overnight incubation with the indicated antibodies (list of antibodies is shown in Table 2). After that, HRP-conjugated antibodies (secondary antibodies) were applied and the intensity signals were detected by an enhanced chemiluminescence (ECL) kit (Merck Millipore, MA, USA) according to the manufacturer’s instructions. β-actin or GAPDH was used as an internal control.

**Table 2:** Antibodies

Antibodies	Source	Identifier
Caspase-3	Cell signaling technology	#9662
Cleaved caspase-3 (Asp175) (5A1E) rabbit mAb	Cell signaling technology	#9664
Caspase-8 (D35G2) rabbit mAb	Cell signaling technology	#4790
Cleaved caspase-8 (Asp387) (D5B2) XP® rabbit mAb (mouse specific)	Cell signaling technology	#8592
RIP3 (B-2) mouse mAb	SANTA CRUZ	sc-374639
Phospho-RIP3 (Thr231/Ser232) (E7S1R) rabbit mAb	Cell signaling technology	#91702
RIP (D94C12) XP® rabbit mAb	Cell signaling technology	#3493
Phospho-MLKL (Ser345) (D6E3G) rabbit mAb	Cell signaling technology	#37333
CYLD (D1A10) rabbit mAb	Cell signaling technology	#8642
TNF-α (D2D4) XP® rabbit mAb (mouse specific)	Cell signaling technology	#11948
Anti-FADD antibody [EPR5030]	Abcam	ab124812
GAPDH antibody	Abways technology	AB0036
NeuN (D4G4O) rabbit mAb	Cell signaling technology	#24307
GFAP (E6N9L) mouse mAb	Cell signaling technology	#34001
Anti-rabbit IgG, HRP-linked antibody	Cell signaling technology	#7074
Anti-mouse IgG (H + L), F(ab')2 fragment (Alexa Fluor® 594 conjugate)	Cell signaling technology	#8890
Anti-rabbit IgG (H + L), F(ab')2 fragment (Alexa Fluor® 488 conjugate)	Cell signaling technology	#4412
IPKine™ HRP, mouse anti-rabbit IgG LCS	Abbkine	A25022

### Immunofluorescence

Mouse brain tissues fixed in 4% paraformaldehyde (PFA) for 24 h were then dehydrated with 30% sucrose for 48 h and sectioned using a cryostat into 5 μm sections. Next, the sections were rewarmed for 30 min, fixed in acetone for 10 min, washed with PBS three times, permeabilized with 0.3% Triton X-100 for 10 min, incubated with a blocking solution (3% bovine serum albumin in PBS) for 1 h and then incubated with indicated antibodies at 4 °C overnight. On the following day, sections were washed three times with PBS (5 min each time), incubated with corresponding secondary antibodies coupled with Alexa Fluor® 488 or Alexa Fluor® 594 at room temperature for 1 h under dark conditions, then washed and mounted with 2-(4-amidinophenyl)-6-indolecarbamidine dihydrochloride (DAPI) (Beyotime, Wuhan, China). Images of fluorescence were captured with an LSM880 confocal laser-scanning inverted microscope (ZEISS, Jena, German).

### Co-immunoprecipitation Assay

Mouse brain tissues were lysed with IP standard lysis buffer (Thermo Fisher scientific, USA) with protease and a phosphatase inhibitor cocktail (1:1000, Thermo Fisher scientific, USA) for 30 min at 4 °C and centrifuged for 15 min at 12,000 rpm. The supernatant was subjected to immunoprecipitation with the indicated antibodies on a rotator for 4 h at 4 °C. Then, 40 μl of agarose-conjugated protein G beads (Roche, Basel, Switzerland) was added, and the mixture was incubated at 4 °C overnight. On the next day, the mixture was washed 5 times (5 min each time) with pre-cooled PBS containing protease and a phosphatase inhibitor cocktail (Thermo Fisher Scientific, USA). Finally, the proteins on the beads were denatured with 1% SDS and the supernatant was subjected to immunoblotting or mass spectrometry (MS) determination.

### Flow Cytometry

To analyse the TNF-α expression level of mouse microglia in response to LSA, N9 cells were seeded in the 12-well cell culture plate and stimulated with LSA (50 μg/ml) for 4 and 24 h. In addition, flow cytometry was performed as follows: cells were washed twice in PBS, treated with fixation and permeabilization solution for 20 min on ice and washed with permeabilized/perm wash buffer (BD Biosciences, CA, USA) according to the manufacturer’s protocol. After that, cells were incubated with primary antibodies and a fluorescein-conjugated secondary antibody followed by flow cytometry analysis. To assess the cell death of neurons stimulated with TNF-α, HT22 cells were cultured in complete medium with TNF-α (10 ng/ml) or TNF-α combined with Z-VAD for 4 h, stained by Annexin V (Invitrogen, USA) and propidium iodide (PI) and washed three times according to the manufacturer’s instructions followed by flow cytometry analysis. The above flow cytometry was conducted on a CytoFLEX flow cytometer (Beckman Coulter, Atlanta, USA) and data were analysed in CytoFLEX.

### Statistical Analysis

In this study, GSEA was conducted via R language (version 3.53, Missouri, USA) and the statistical analysis of the difference was performed with the use of GraphPad Prism 7.00 (San Diego, CA, USA). Data were presented as the means ± SDs. Student's *t* tests were used to evaluate significant difference between two independent groups and a *p* value lower than 0.05 was considered statistically significant.

## Results

### GSEA Reveals TNF-α-Related Apoptosis and Necroptosis in the Mouse Brain Upon AC Infection

**Fig. 1:**
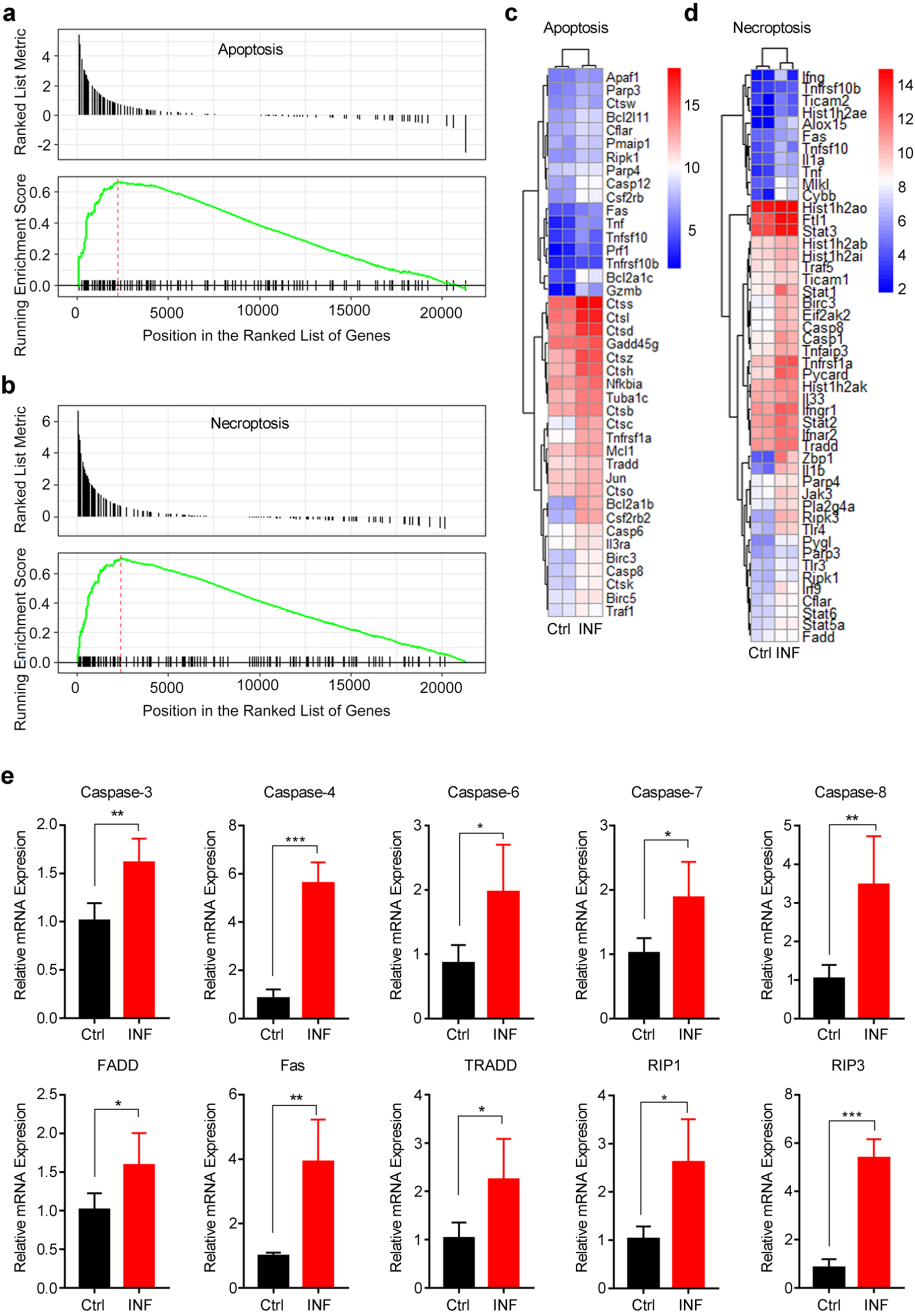
GSEA reveals the occurrence of apoptosis and necroptosis in mouse brains upon AC infection. **a**, **b** The brain tissues of normal control and AC-infected mice (*n* = 2 mice/group) were subjected to a gene expression array, and the DEGs between the two groups were further analysed by GSEA, which indicated a significant association of AC infection with apoptosis (**a**), necroptosis (**b**) and signalling pathways. **c**, **d** Heatmap displayed the relative mRNA expression level of apoptosis and necroptosis pathway-related genes enriched in **a**, **b**. **e** The DEGs involved in apoptosis and necroptosis were dramatically upregulated in AC-infected mice (*n* = 4) compared with in normal control mice (*n* = 4). *p < 0.05, **p < 0.01, ***p < 0.001 (student’s *t* test). *AC Angiostrongylus cantonensis*

**Fig. 2:**
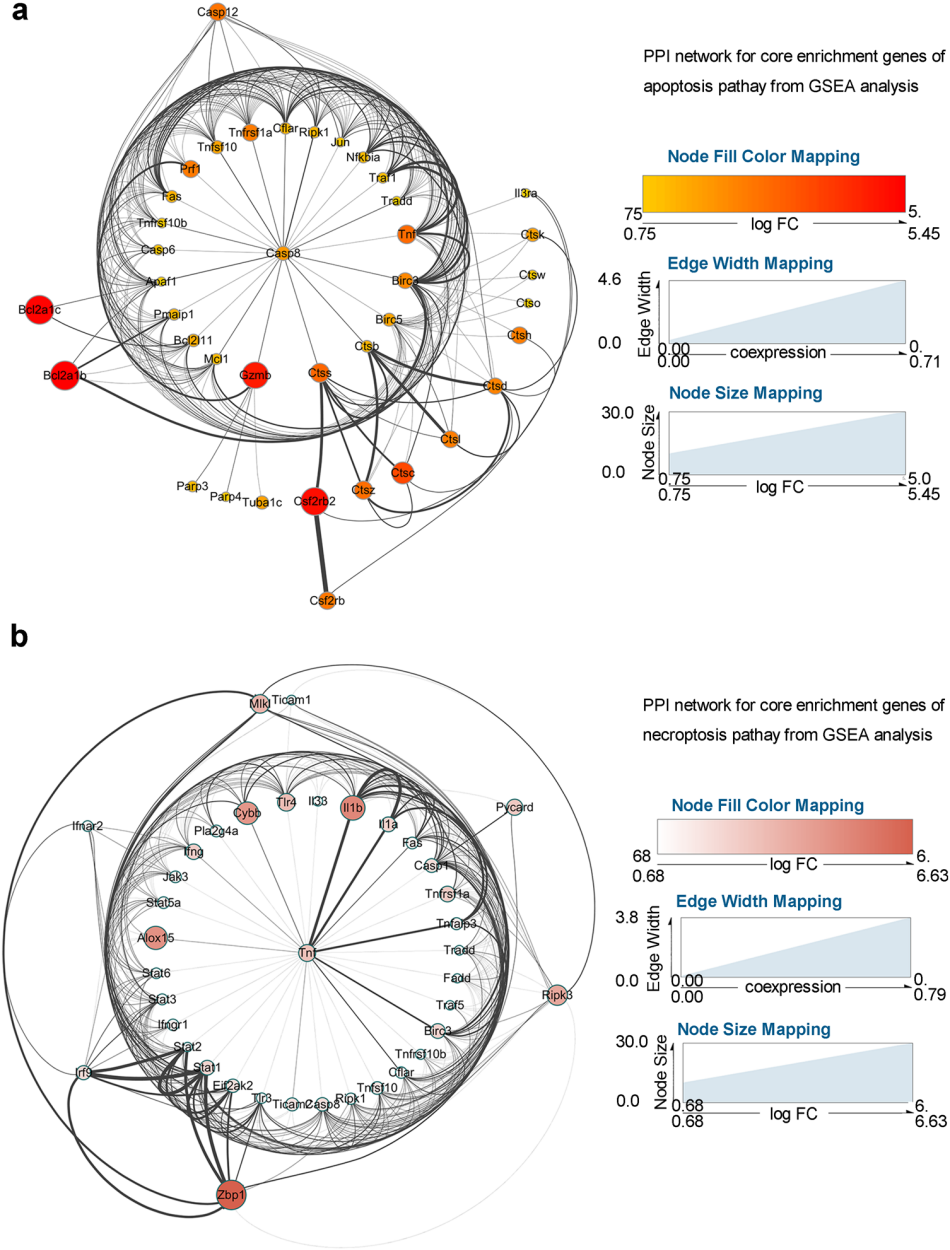
The PPI network for the core enriched genes related to the apoptosis and necroptosis pathways. **a**, **b**. PPI network analysis for the core enriched genes of GSEA (as shown in Fig. 1c, d) showed that caspase-8 and TNF-α were the hub genes for the apoptosis and necroptosis pathways in mouse brains infected by AC, respectively. *AC Angiostrongylus cantonensis*

In our previous study, the dramatic apoptosis and necroptosis levels were discovered in the parenchyma and hippocampus of the mouse brain upon AC infection (Mengying et al. 2017). To further investigate the underlying mechanisms of apoptosis and necroptosis in the mouse central nervous system (CNS) induced by AC, we first performed gene expression profiles for whole-brain tissues of AC-infected and normal control mice (*n* = 2 mice/each group) and then GSEA was applied via R language 3.53 to confirm the occurrence of apoptosis and necroptosis. First, we confirmed that all the mice in INF and ACE group successfully developed into angiostrongyliasis after AC infection. Similar to human, mice developed into angiostrongyliasis exhibited impaired neurological function along with imbalanced walking state (Lai et al. 2020). After infection with twenty third-stage infectious AC larvae by oral gavage, mice were monitored the balance capacity by observing the walking balance ability every day. All the mice displayed impaired balance capacity with the symptom of unbalanced walking at eighteen days post infection. Most importantly, immature AC larvae were found on the cerebral surface of all mice in INF and ACE group, providing the most straightforward evidence of successful infection with AC. Next, we performed data analysis for expression profiles and demonstrated that the gene sets of apoptosis and necroptosis in the mouse brains infected by AC were significantly enriched compared with those in the normal control mouse brains (Fig. 1a, b). In total, there were 41 and 48 obviously upregulated core genes markedly enriched in the apoptosis and necroptosis pathway, respectively, as shown in the heatmap (Fig. 1c, d). These core genes were principally associated with the death receptor signalling pathway, and the relative mRNA expression levels of 10 critical genes were checked by RT-qPCR. The results exhibited that the mRNA levels of TRADD, RIP1, caspase-3, caspase-6, and caspase-7 were upregulated twofold compared with those of the normal control; moreover, the mRNA levels of Fas, caspase-8, caspase-4 and RIP3 were fourfold higher than in the normal control (Fig. 1e), which is concordant with the GSEA results. To explore the interactions and hub genes among these core genes of apoptosis and necroptosis, we constructed a PPI network by Cytoscape software and found that caspase-8 and TNF-α shared most of the interactions in the apoptosis and necroptosis network, respectively, which demonstrated that caspase-8 was the hub gene for the apoptosis pathway (Fig. 2a) while TNF-α acted as the hub gene for the necroptosis pathway (Fig. 2b). Furthermore, Caspase-8 was reported to serve as a downstream gene regulated by TNF-α (Zheng et al. 2016). Collectively, these above data indicated that the apoptosis and necroptosis in mouse brains upon AC infection was closely related to TNF-α.

### TNF-α Induces Apoptosis and Necroptosis During AC Infection

**Fig. 3:**
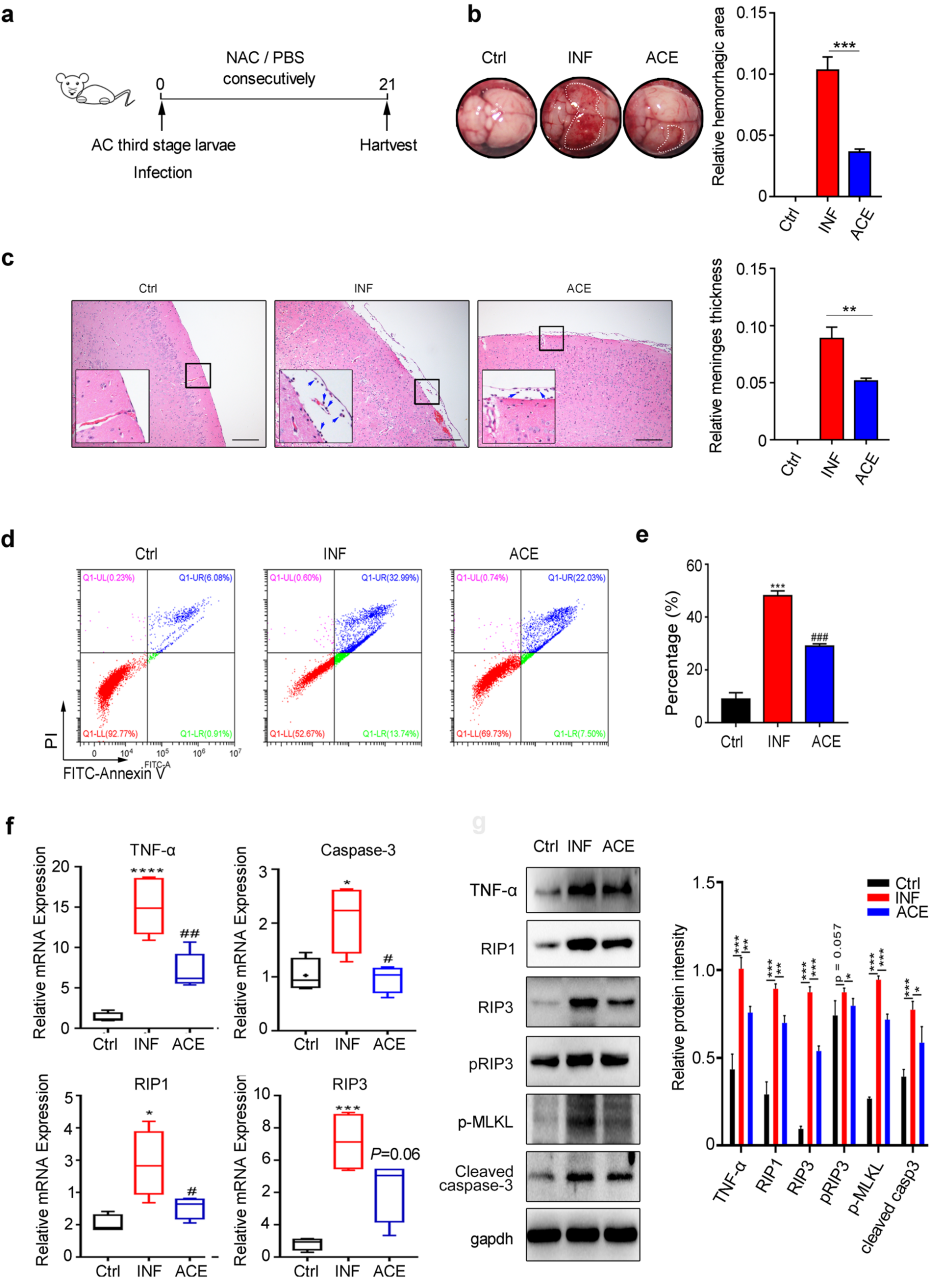
Inhibition of TNF-α alleviates the inflammation and cell death of mouse brains with AC infections. **a** Mice (*n* = 4 per group) were consecutively administered ACE or PBS after AC infection. **b** Representative images of brain tissues in mice from three groups (left) and quantitative analysis for relative haemorrhagic surface area (right). **c** Pathological morphology of brain tissues was evaluated by H&E staining (left) and quantitative analysis for relative meninges thickness in H&E staining (right). **d** Flow cytometry analysis was used to analyse the cell death of mouse brains. **e** Percentage of dead cells showed in **d**. **f** mRNA levels of TNF-α, RIP1, RIP3 and Caspase-3 were detected after ACE treatment (*n* = 4 mice/group). **g** Protein levels of RIP1, RIP3, pRIP3, pMLKL and cleaved caspase-3 in mouse brains of the three groups were examined by western blot (left) and quantitative analysis for protein intensity (right, normalized to GAPDH). *ACE* acetylcysteine. *p < 0.05, ***p < 0.001. ****p < 0.0001 compared to the Ctrl group, #*p* < 0.05, ##*p* < 0.01 compared to the INF group (student’s *t* test)

To further investigate whether TNF-α induced apoptosis and necroptosis during AC infection, we conducted a rescue assay in vivo. First, several reported selective inhibitors (Man et al. 2009; Peristeris et al. 1992; Sampaio et al. 1991) of TNF-α (thalidomide, apremilast and acetylcysteine) were tested in our model to evaluate inhibition effects on TNF-α production, among which acetylcysteine (ACE) exhibited the best inhibitory efficacy. Therefore, ACE was chosen as the specific inhibitor of TNF-α in the present study. Next, we divided mice into three groups (normal control: Ctrl; AC infection: INF; AC infection combined with acetylcysteine treatment: ACE), and the mice of INF and ACE groups were infected with AC. Additionally, the mice of ACE and INF groups were separately treated with acetylcysteine and PBS daily (Fig. 3a). Next, the mice of the three groups were sacrificed at 21 days post infection, and brain tissues were collected for haemorrhagic focus observation and pathological and molecular biological analyses. The results illustrated that AC infection caused severe haemorrhage in mouse brain tissues compared with normal control while TNF-α inhibitor treatment could reverse the effect (Fig. 3b), implying that TNF-α inhibitor could reduce the inflammatory response induced by AC. In addition, pathological configuration in the mouse brain displayed more thick meninges and more infiltrated inflammatory cells after AC infection, and these characteristics could be obviously attenuated with ACE treatment (Fig. 3c). To explore whether the cell death of brain cells was simultaneously alleviated, we performed flow cytometry and observed that the number of dead cells in the mouse brains of the ACE group were dramatically decreased compared with that in the INF group (Fig. 3d, e). In line with the above result, the mRNA level of a specific marker for apoptosis and necroptosis was increased after AC infection but could be reversed by ACE treatment (Fig. 3f). Moreover, the levels of critical proteins involved in apoptosis (RIP1 and cleaved caspase-3) and necroptosis (RIP3, pRIP3 and pMLKL) in the mouse brains also displayed the expected change upon AC infection and TNF-α inhibitor treatment, as demonstrated by immunoblotting (Fig. 3g). Hence, TNF-α was confirmed to induce the apoptosis and necroptosis caused by AC.

### TNF-α Triggers RIP1/FADD/Caspase-8-Mediated Apoptosis of Astrocytes During AC Infection

**Fig. 4:**
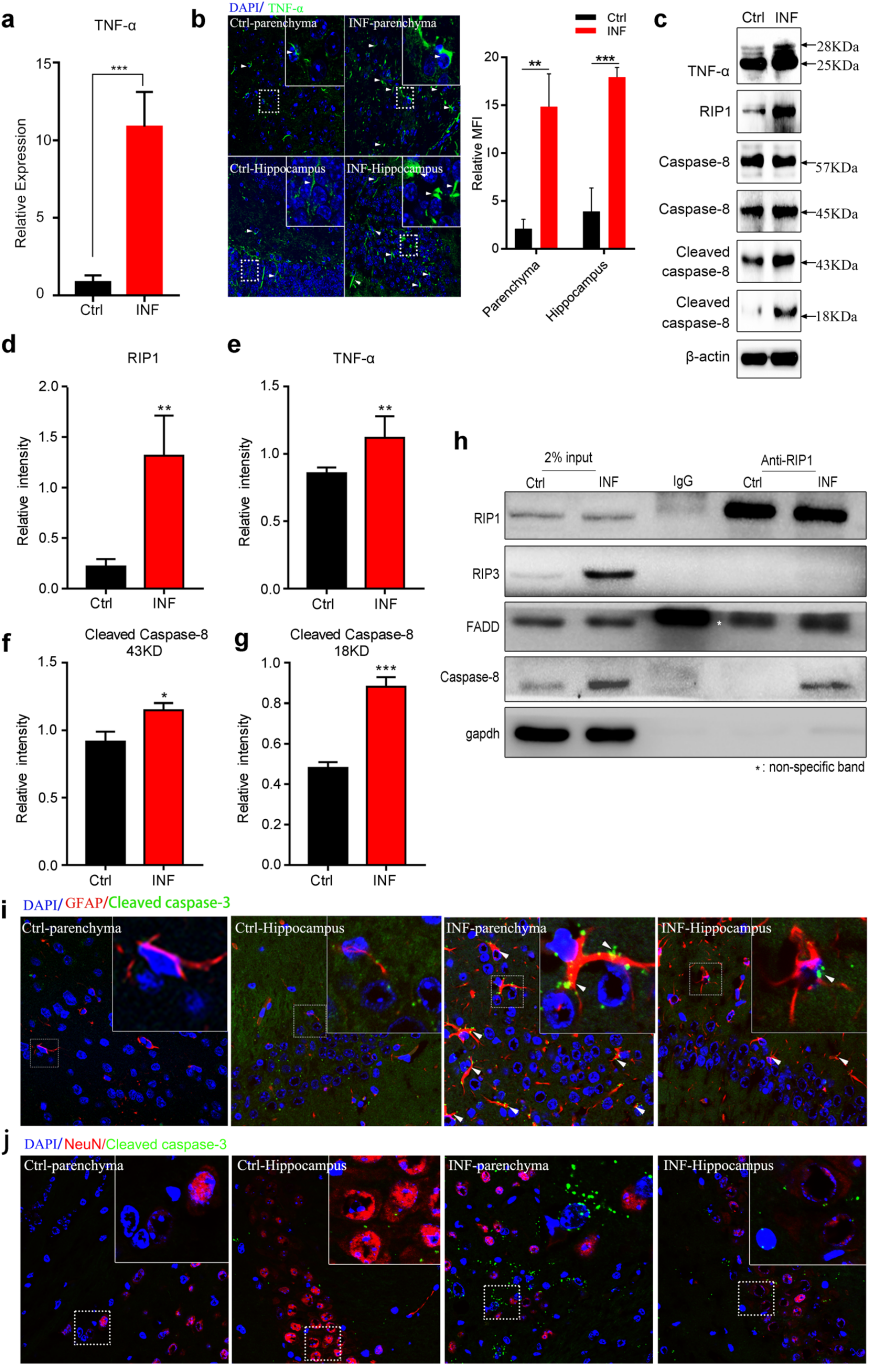
TNF-α triggers RIP1/FADD/caspase-8-mediated apoptosis of astrocytes in mouse brains with AC infection. **a** Relative mRNA level of TNF-α in mouse brains upon AC infection was significantly higher than that in the control group. **b** TNF-α protein expression level in the mouse brain post infection was remarkably elevated, as revealed by immunofluorescence (left) and relative mean fluorescence intensity (MFI, right). **c** The lysates of brain tissues from normal control mice or mice post infection were subjected to western blot to determine the protein levels of genes related to the apoptosis signalling pathway (TNF-α, RIP1, caspase-8 and cleaved caspase-8). **d**–**g** The protein expression levels in **c** were quantified via density analysis. **h** Mice were infected by AC for 21 days and then the lysates of mouse brain tissues were immunoprecipitated with an anti-RIP1 antibody or an IgG antibody and the precipitated complexes were separately analysed by immunoblotting with antibodies against RIP1, RIP3, FADD, caspase-8 and GAPDH. **i** Cleaved caspase-3 and GFAP (specific marker of astrocytes) were co-localized, as shown by immunofluorescence. **j** Cleaved caspase-3 and NeuN (specific marker of neurons) showed no co-localization, as shown by immunofluorescence. *p < 0.05, **p < 0.01, ***p < 0.001 (student’s *t* test). *AC Angiostrongylus cantonensis*, *Ctrl* normal control, *INF* infected by *Angiostrongylus cantonensis*

TNF-α acts as a pro-inflammatory cytokine that regulates various signalling pathways such as inflammation (Zelova and Hosek 2013), apoptosis (Naimi et al. 2018) and necroptosis (Gunther et al. 2011). As described above, TNF-α induced apoptosis in mouse brains with AC infections. To demonstrate the specific molecular basis of TNF-α regulating apoptosis in this study, we first detected the transcript level of TNF-α using RT-qPCR, and the result showed that AC-infected mouse brain tissues exhibited a much higher TNF-α mRNA level than normal control mice (Fig. 4a). We further performed immunofluorescence with an antibody against TNF-α in the brain parenchyma and hippocampus of mice infected by AC and normal control mice to check the protein expression level of TNF-α. Not unexpectedly, the fluorescence signal in the AC-infected mouse brain parenchyma and hippocampus was significantly enhanced compared with that in the normal control mouse brain (Fig. 4b), indicating that the TNF-α protein was upregulated upon AC infection. TNF-α could induce pathological and physiological apoptosis by activating various downstream genes, including RIP1, FADD and caspase-8. To identify the specific TNF-α signalling pathway triggering apoptosis in our study, immunoblotting was conducted to detect the expression level of downstream genes of TNF-α, and we found that the grey value of TNF-α (25 kD and 28 kD), RIP1 and cleaved caspase-8 (18 kD and 43 kD) substantially accumulated during AC infection, while for total caspase-8, no significant change was observed (Fig. 4c). Furthermore, density analysis for western blot in Fig. 4c revealed that the protein levels of RIP1, TNF-α and cleaved caspase-8 (43 kD and 18 kD) markedly increased (Fig. 4d-g), of which, RIP1 was enhanced fourfold, indicating that RIP1 could be the main regulator of apoptosis in our study. RIP1 was reported to interact with FADD and caspase-8 under TNF-α stimulation to induce cell apoptosis (Zheng et al. 2016). To confirm whether the TNF-α/RIP1/FADD/caspase-8 signalling pathway was involved in cell apoptosis in our disease model, we performed co-immunoprecipitation using an anti-RIP1 antibody and an IgG control antibody for the lysates of the whole-brain tissues from AC-infected mice and normal control mice. With an immunoblotting analysis for the precipitated complex, RIP1 was found to interact directly with FADD and caspase-8 instead of RIP3; moreover, the interaction between RIP1 and caspase-8 was heightened after AC infection (Fig. 4h), which indicated that TNF-α induced cell apoptosis in our study by activating the RIP1/FADD/caspase-8 pathway. As reported, Caspase-8 functioned in apoptosis progression through Cleaved caspase-3 (executioner of apoptosis), and thus, cleaved caspase-3 is usually selected as the specific marker for apoptosis (Zheng et al. 2016). Next, to determine the specific cell type during apoptosis in mouse brains with AC infections, we carried out immunofluorescence in the mouse brain parenchyma and hippocampus with an anti-cleaved caspase-3 antibody and an antibody against a specific cell marker such as GFAP (specific marker for astrocytes) to track a specific cell type in brain tissue. The results show that only GFAP could be co-localized with cleaved caspase-3 and that the co-localization between GFAP and cleaved caspase-3 was elevated during AC infection (Fig. 4i), indicating that astrocytes were the main cell type undergoing apoptosis in mouse brains after infection with AC. Moreover, we also examined the apoptosis of other cerebral cells like neurons (Fig. 4j) but failed to observe any significant co-localization of cleaved caspase-3 and NeuN, as shown in Fig. 4j, suggesting that no obvious apoptosis occurred in neurons upon AC infection in mice. Altogether, TNF-α elicited RIP1/FADD/caspase-8-mediated apoptosis of astrocytes upon AC infection.

### TNF-α Triggers RIP3/MLKL-Mediated Necroptosis of Neurons Upon AC Infection

**Fig. 5:**
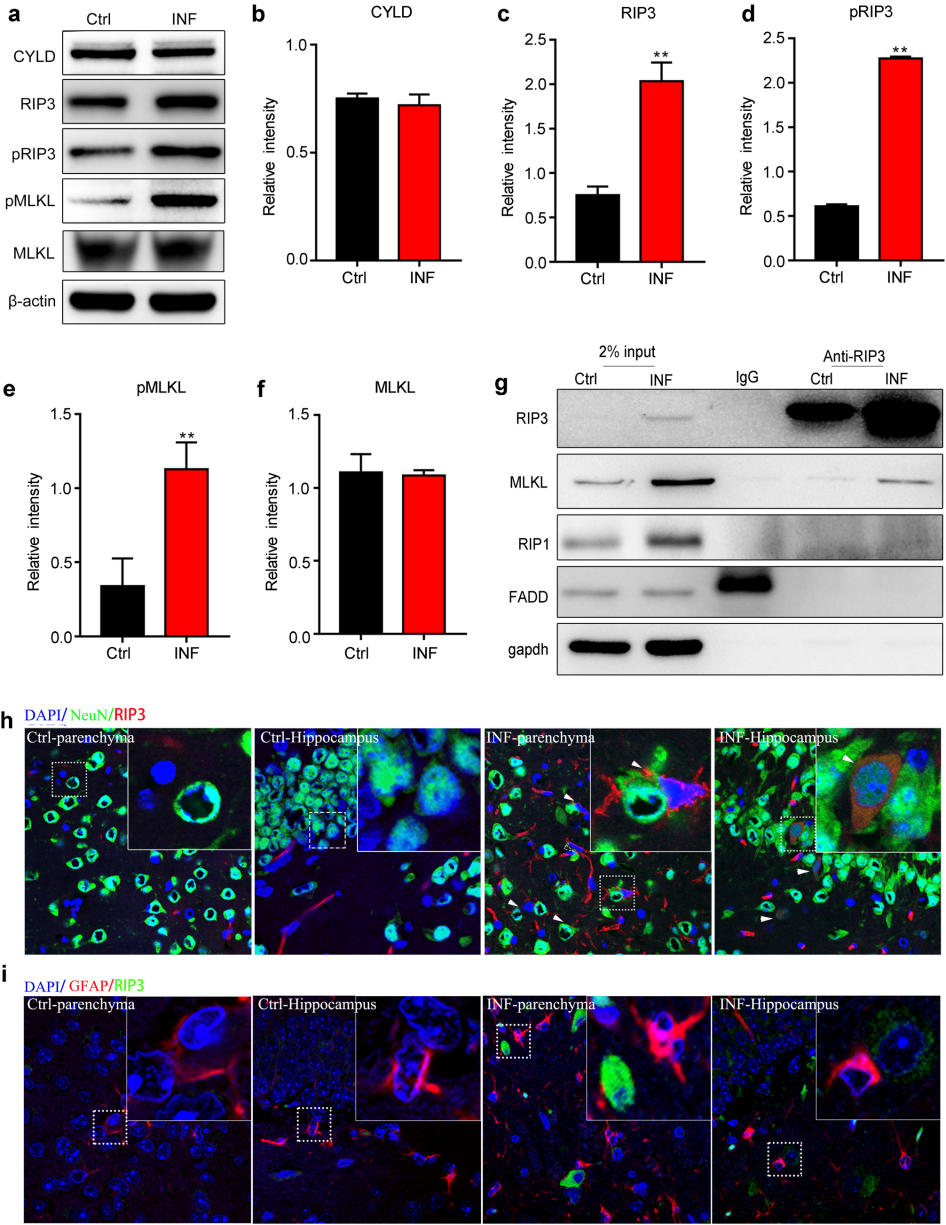
TNF-α triggers RIP3/MLKL-mediated necroptosis of neurons in mouse brains infected by AC. **a** The lysates of brain tissues from normal control mice or mice post infection were subjected to western blot to determine the protein levels of genes related to the necroptosis pathway (RIP3, pRIP3, CYLD and pMLKL). **b**–**f** The protein levels in **a** were quantified via density analysis. **g** Mice were infected by AC for 21 days and then the lysates of mouse brain tissues were immunoprecipitated with an anti-RIP3 antibody or an IgG antibody, and the precipitated complexes were separately analysed by immunoblotting with antibodies against RIP3, MLKL, RIP1, FADD and GAPDH, respectively. **h** RIP3 (specific marker of necroptosis) was co-localized with NeuN (specific marker of neurons), as displayed by immunofluorescence. **i** RIP3 (specific marker of necroptosis) exhibited no co-localization with GFAP (specific marker of neurons), as showed by immunofluorescence. *p < 0.05, **p < 0.01, ***p < 0.001 (student’s *t* test). *AC Angiostrongylus cantonensis*, *Ctrl* normal control, *INF* infected by *Angiostrongylus cantonensis*

In addition to apoptosis, TNF-α additionally induced necroptosis in this study. The signalling pathway involved in necroptosis comprises diverse critical proteins, including CYLD, RIP3, pRIP3 and pMLKL. To verify the existence of necroptosis in our model, we first checked whether the protein levels of these critical proteins related to necroptosis varied in the AC infection group compared with that in the normal control group. With immunoblotting analysis, we found that the grey value of RIP3, pRIP3 and pMLKL was much more intensive in the brain tissues of AC-infected mice than those of normal control (Fig. 5a). Quantitative analysis for the western blot in Fig. 5a revealed that AC infection could significantly elevate the protein levels of RIP3, pRIP3 and pMLKL, but not that of CYLD (Fig. 5b–e). As reported previously, the RIP1/RIP3/MLKL complex was involved in the necroptosis induced by TNF-α (Hu et al. 2020). To explore whether TNF-α induced necroptosis through the same mechanism in our study, a co-immunoprecipitation assay was performed with an anti-RIP3 antibody and an IgG antibody followed by immunoblotting analysis for the precipitated complex. Unexpectedly, the result delineated that RIP3 could bind to MLKL but to neither RIP1 nor FADD (Fig. 5f–g), which was distinct from the reported signalling pathway of necroptosis induced by TNF-α in a reported model. Moreover, to confirm the specific cell type under necroptosis in mouse brains upon AC infections, we implemented immunofluorescence in the mouse brain parenchyma and hippocampus with antibodies against RIP3 (specific marker for necroptosis) and NeuN (specific marker for neurons) to track a specific cell type in brain tissues. We found that NeuN was overlapped with RIP3 and that the co-localization of NeuN and RIP3 was absent in normal control mouse brain tissues (Fig. 5h), indicating that neurons were undergoing necroptosis in mouse brain tissues. Moreover, we also checked the necroptosis of other non-neuronal cells like astrocytes (Fig. 5i) but failed to find any co-localization between RIP3 and GFAP, indicating that no necroptosis occurred in astrocytes upon AC infection. The above results indicate that TNF-α elicited RIP3/MLKL-mediated necroptosis of neurons in our study.

### TNF-α Secreted by Microglia Promotes Necroptosis of Neurons

**Fig. 6:**
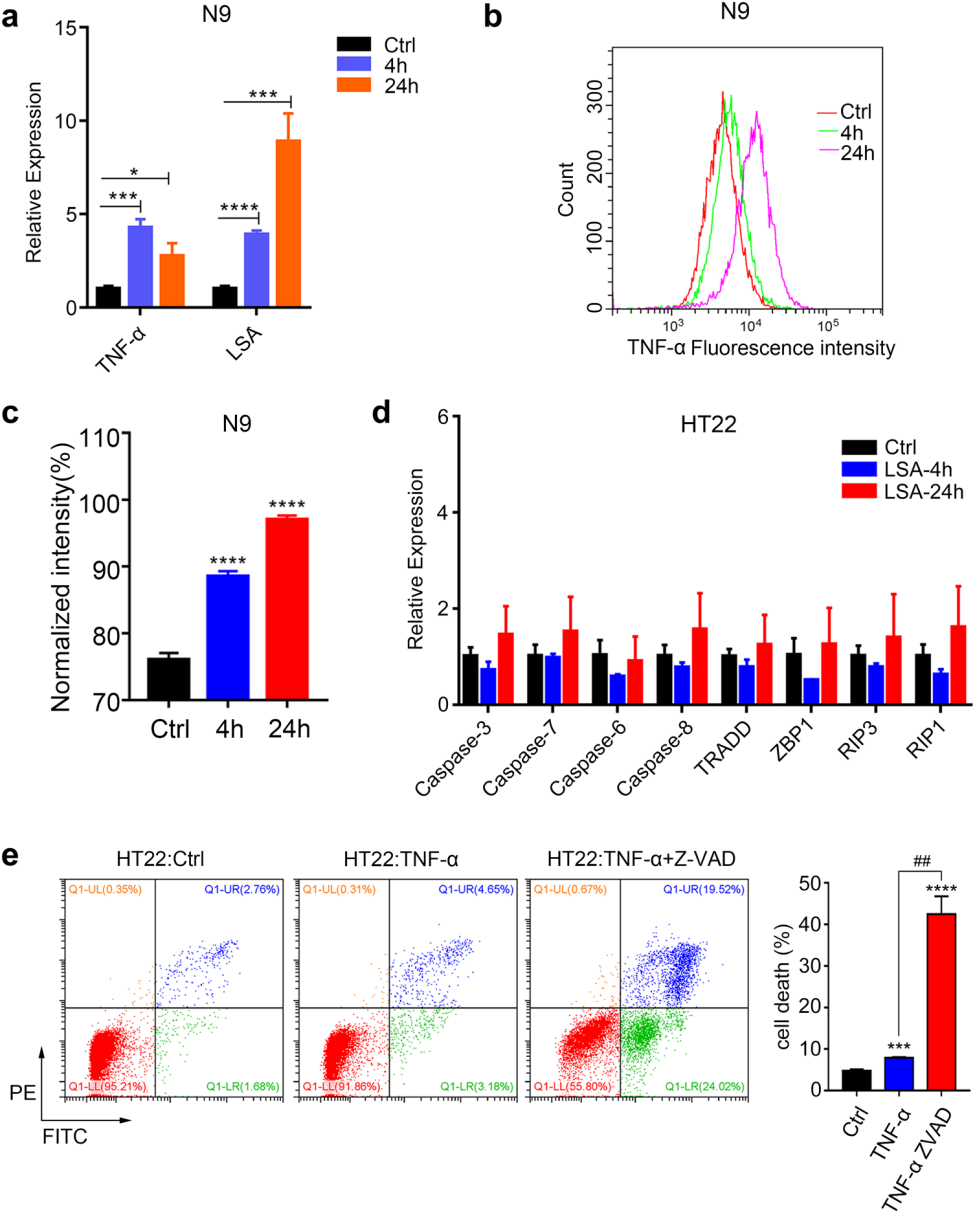
TNF-α secreted by microglia promotes necroptosis of neurons. **a** The transcription level of TNF-α in the N9 cell line treated with TNF-α or LSA was much higher than that in control cells. **b** The protein level of TNF-α in the N9 cell line treated with LSA was significantly higher than that in the control group, as shown by flow cytometry. **c** Quantitative result of **b** showed the protein level of TNF-α in N9. **d** The mRNA expression levels of genes related to apoptosis and necroptosis in the mouse hippocampus neuron cell line HT22 treated with LSA for 4 or 24 h showed no significant change compared to that in control cells, as exhibited by RT-qPCR. **e** The cell death of the HT22 cell line treated with TNF-α or TNF-α combined with Z-VAD (inhibitor for caspases) was analysed by flow cytometry with triple independent repeats. *p < 0.05, ***p < 0.001, ****p < 0.0001 (student’s *t* test). *LSA* larvae soluble antigen

As reported previously, AC is a sort of parasite that stays in mouse brain tissues and dies to form the LSA that stimulates the brain cells to exert a drastic inflammatory response related to TNF-α (Paouri and Georgopoulos 2019). In this study, TNF-α was confirmed to provoke necroptosis of neurons during AC infection. However, the cell type in the mouse brain that secreted TNF-α to promote necroptosis of neurons upon AC infection was still unclear. Microglia were the only resident immune cells and the main source of inflammatory cytokines in mouse brain (Hansen et al. 2018). Thus, we surmised that microglia likely secreted TNF-α upon LSA stimulation. To verify our conjecture, we treated mouse microglia (N9 cell line) in vitro with TNF-α (10 ng/ml, as a positive control) and LSA (50 μg/ml, prepared under aseptic conditions) for 4 and 24 h to detect the expression level of TNF-α. With RT-qPCR, we found that the mRNA level of TNF-α was upregulated by 4.5-fold and ninefold at 4 and 24 h after LSA treatment (Fig. 6a). Simultaneously, the protein level of TNF-α was significantly boosted (Fig. 6b–c) as exhibited by flow cytometry. Next, we continued to explore whether necroptosis of neurons was directly induced by LSA from dead parasites or TNF-α secreted by activated microglia. First, mouse neurons (HT22 cell line) were treated by LSA (50 μg/ml) for 4 and 24 h in vitro but failed to provoke a notable upregulation of genes involved in cell death, indicating that LSA could not directly induce the cell death of neurons in our model (Fig. 6d). However, when HT22 cells were stimulated with TNF-α (10 ng/ml) for 4 h, more dead neurons were observed in the treated group (Fig. 6e) than in the normal control group (7.8% vs 4.44%). In addition, when we treated HT22 cells with TNF-α combined with Z-VAD (inhibitor of caspase family), the cell death of neurons was more pronounced (Fig. 6e) than with TNF-α treatment (43.54% vs 7.8%), revealing that TNF-α could elicit necroptosis of neurons but not apoptosis. Taken together, these results demonstrate that AC infection led to TNF-α secretion by activated microglia to mediate necroptosis of neurons.

## Discussion

Programmed cell death such as apoptosis, necroptosis and autophagy of brain cells plays a pivotal role in neurodegenerative diseases such as Alzheimer's disease, Parkinson's disease, Huntington's disease and irreversible neurological impairment caused by parasitic infection (Yu and He 2016; Yuan et al. 2019). For non-parasitic neurodegenerative disease, the underlying mechanism of cell death in the brain has been well studied. For example, apoptosis of neurons during Alzheimer's disease progression could be induced by PGD2 which stimulated expression of BIK and suppressed expression of ARRB1 (Guo et al. 2017). Neuron autophagy in Parkinson's disease could be regulated by MIR-124 targeting Bim (Wang et al. 2016) while apoptosis of neurons in Huntington's disease was induced by NMDAR-mediated excitotoxicity (Fernandes et al. 2007). As has been reported, an AC infection could cause neurodegenerative symptoms in a mouse model (Ji et al. 2017), and in our previous study, we fully confirmed that AC could induce apoptosis and necroptosis of mouse brain cells (Mengying et al. 2017). However, to date, there are no studies elucidating the specific molecular basis.

In this study, we intended to explore the explicit mechanisms of apoptosis and necroptosis in the mouse brain during AC infection. To this end, we first performed GSEA and PPI network construction analysis on the expression profile of mouse brains with or without AC infection and finally identified TNF-α as the hub gene in the regulatory network of apoptosis and necroptosis; these results imply that TNF-α likely plays a central role in the cell death of mouse brain cells with AC infection. To validate this conjecture, we conducted a rescue assay in vivo*,* and the result indicated that TNF-α was upregulated after AC infection, which is concordant with the results of a previous study (Chen and Lai 2007), and was downregulated after acetylcysteine treatment. Accordingly, inhibition of TNF-α could reverse the effect of AC infection on inflammation and cell death in the mouse brain, which verified that TNF-α induced apoptosis and necroptosis during AC infection.

Next, we aimed to further explore the concrete molecular network underlying TNF-α-induced apoptosis and necroptosis in our study. For TNF-α-induced apoptosis, many studies have demonstrated that the downstream signalling pathway was mainly the RIP1/FADD/Caspase-8 axis which was characterized by the formation of the RIP1/FADD/Caspase-8 complex by cleaving Caspase-8 and then activating Caspase-3 to execute apoptosis (Abhari et al. 2013; Hu et al. 2020; Ikner and Ashkenazi 2011; Long et al. 2019). Therefore, we first investigated whether the same signalling axis was involved in the apoptosis observed in our model. With a co-immunoprecipitation assay for mouse brain tissues, we found that in this study, RIP1 could directly bind to FADD and Caspase-8 to form a complex, which was in line with the previously reported signalling axis (Abhari et al. 2013; Hu et al. 2020; Ikner and Ashkenazi 2011; Long et al. 2019). Furthermore, the interaction between RIP1 and FADD/Caspase-8 was notably enhanced after AC infection. In addition, we performed immunofluorescence to identify astrocytes undergoing apoptosis and this was similar to the outcome of astrocytes in rats infected with AC (Zhou et al. 2019). For TNF-α-induced necroptosis, previous studies indicated that the downstream signalling axis was the RIP1/RIP3/MLKL pathway characterized by the formation of the RIP1/RIP3/MLKL complex (Wang et al. 2019; Zhang et al. 2018). To elucidate the specific signalling pathway regulated by TNF-α in our study, we also conducted co-immunoprecipitation using mouse brain tissues and unexpectedly discovered that RIP3 merely bound to MLKL, which was distinct from the canonical signalling axis described above. Similarly, the RIP3/MLKL complex was also discovered to be involved in necroptosis due to murine cytomegalovirus and influenza A virus infections (Thapa et al. 2016; Upton et al. 2019); in the absence of RIPK1, RIP3/MLKL triggers necroptosis of cells induced by IFNs (Ingram et al. 2019; Upton et al. 2019). Thus, we speculate that there should be some similar mechanism leading to necroptosis between parasite and virus infections. Finally, we confirmed necroptosis of neurons induced by AC via immunofluorescence. Although through the in vivo experiments above, we generally could draw the conclusion that TNF-α drove apoptosis of astrocytes and necroptosis of neurons after AC infection, inclusion of another group of mice treated with TNF-α inhibitor will better confirm this conclusion.

As a pro-inflammatory cytokine, TNF-α is mainly secreted by cells involved in the inflammatory response, including macrophages, natural killer (NK) cells, and lymphocytes (Wang et al. 2012, 2018). As is known, in brain tissue, microglia act as the sole resident immune cell, thus, we surmised that the high level of TNF-α was produced by microglia upon AC infection. To verify this assumption, we performed an in vitro assay to stimulate N9 (mouse microglia cell line) with LSA, and the result confirmed that AC infection could induce microglia to secret TNF-α. Recently, microglial TNF-α was reported to be elevated under pathological conditions, which was in concordance with our results (Borrajo et al. 2014; Lewitus et al. 2016). However, the increased microglial TNF-α had diverse functions, including suppressing neuronal plasticity (Lewitus et al. 2016) and mediating dopaminergic degeneration. Dissimilarly, in this study, upregulation of microglial TNF-α could induce RIP3/MLKL-mediated necroptosis of neurons, which expanded our knowledge of the role of microglia.

## Conclusion

In summary, the present study first illustrated that the TNF-α secreted by microglia during AC infection induced RIP1/FADD/caspase-8-mediated apoptosis of astrocytes and RIP3/MLKL-mediated necroptosis of neurons. Our research comprehensively elucidated the mechanism of cell death after AC infection and provided new insight into targeting TNF-α signalling for therapeutic strategies during CNS injury.

## Data Availability

The data supporting the conclusions of this article are included within the article and its additional files.

## Abbreviations

AC: *Angiostrongylus cantonensis*
ACE: Acetylcysteine
BCA: Bicinchoninic acid
CNS: Central nervous system
Ctrl: Normal control
DEGs: Differentially expressed genes
dpi: Days post infection
ECL: Enhanced chemiluminescence
GSEA: Gene set enrichment analysis
INF: Infected by *Angiostrongylus cantonensis*
LSA: Larvae soluble antigen
MS: Mass spectrometry
PFA: Paraformaldehyde
PPI: Protein–protein interaction
RIP3: Receptor interacting kinase protein3
RT-qPCR: Quantitative reverse transcription-polymerase chain reaction
TNF-α: Tumour necrosis factor-α
